# Magnitude and correlates of bird collisions at glass bus shelters in an urban landscape

**DOI:** 10.1371/journal.pone.0178667

**Published:** 2017-06-01

**Authors:** Christine M. Barton, Corey S. Riding, Scott R. Loss

**Affiliations:** Department of Natural Resource Ecology & Management, Oklahoma State University, Stillwater, Oklahoma, United States of America; Auburn University, UNITED STATES

## Abstract

Wildlife residing in urban landscapes face many human-related threats to their survival. For birds, collision with glass on manmade structures has been identified as a major hazard, causing hundreds of millions of avian fatalities in North America every year. Although research has investigated factors associated with bird-glass collision mortality at buildings, no prior studies have focused on bird fatalities at glass-walled bus shelters. Our objectives in this study were to describe the magnitude of bird-bus shelter collisions in the city of Stillwater, Oklahoma and assess potential predictors of collision risk, including characteristics of shelters (glass area) and surrounding land cover (e.g., vegetative features). We surveyed for bird carcasses and indirect collision evidence at 18 bus shelters over a five-month period. Linear regression and model selection results revealed that the amount of glass on shelters and the area of lawn within 50 m of shelters were both positively related to fatal bird collisions; glass area was also positively associated with observations of collision evidence on glass surfaces. After accounting for scavenger removal of carcasses, we estimate that a minimum of 34 birds are killed each year between May and September by collision with the 36 bus shelters in the city of Stillwater. While our study provides an initial look at bird fatalities at bus shelters, additional research is needed to generate a large-scale estimate of collision mortality and to assess species composition of fatalities at a national scale. Designing new bus shelters to include less glass and retrofitting existing shelters to increase visibility of glass to birds will likely reduce fatal bird collisions at bus shelters and thus reduce the cumulative magnitude of anthropogenic impacts to birds in cities.

## Introduction

Animals inhabiting human-dominated landscapes face a wide variety of threats associated with changes to habitat, biota, and the built environment. For birds, major hazards that directly cause fatalities in anthropogenic landscapes include predation by cats [1,2] and collision with manmade structures and vehicles [3,4,5]. Bird collisions with glass windows in particular have recently received increased research, conservation, and policy attention, and between 365 and 988 million birds are estimated to be killed annually by window strikes in the U.S. alone [6]. Although building windows have been the primary focus of collision research in urban landscapes, other structures with large amounts of glass, including zoo enclosures [7] and elevated walkways [8,9], also cause avian deaths by collision. However, to date, no studies have assessed the risk that glass-walled bus shelters pose to birds.

Understanding factors affecting bird-glass collision rates is crucial to developing solutions that minimize avian mortality. Studies of bird-window collisions have identified several correlates of collision rates, including habitat features [8,10,11], window area [8,12], seasonality [8,11,13], time of day [10], and avian density [12]. Similar factors may also influence rates of bird collisions at bus shelters, such as amount of glass, amount and proximity of nearby vegetation, and amount of undeveloped green space in the surrounding landscape. Vegetation in urbanized areas offers resources for nesting, food, and shelter, but also draws birds closer to human structures [14,15] and can elevate collision risk [11,8]. Additionally, the proportion of undeveloped land surrounding buildings is positively related to window collision rates [12], suggesting that glass structures in suburban areas and near extensive green spaces are likely to be more collision-prone. Because bus shelters are generally found in a variety of urban landscape contexts, including suburban neighborhoods, downtown areas, and inner-cities, similar patterns of collision risk in association with urbanization intensity may be expected.

The physical properties of glass also influence bird collision rates. Birds strike glass because they are unable to perceive it as a barrier [4,16], and this perception failure occurs either when reflective glass mirrors the surrounding environment or transparent glass creates a see-through effect by which birds are drawn to indoor plants or features on the structure’s opposite side. The see-through effect is especially problematic for structures constructed largely of glass (e.g., glass walkways [8,9,17]), and the typical architecture of glass bus shelters (i.e., two or more facades entirely constructed of glass) is likely to similarly mislead birds and cause high collision rates.

Despite incidental reports of bird collision fatalities at glass-walled bus shelters, no studies to date have formally recognized and rigorously researched this phenomenon. We sought to describe and evaluate the magnitude of bird collision mortality at bus shelters by conducting formal bird collision monitoring surveys in the Stillwater, Oklahoma urban area. Our objectives were to identify associations between fatality rates and characteristics of shelters and their surroundings, to estimate the number of fatal bird collisions at bus shelters in our study area, and to suggest future research avenues and place bird-bus shelter collisions within the broader context of avian collisions with glass structures.

## Materials and methods

### Study area

We monitored bus shelters in Stillwater, Oklahoma, USA (36.1156° N, 97.0584° W), an urban area with a human population of roughly 47,000 and lying in the Cross Timbers region, a transitional zone consisting of prairie, shrubland, and oak savanna/woodland between the eastern deciduous forests to the east and Great Plains to the west. To select bus shelters for our study, we first used aerial imagery (Google Earth) and photographs (Street View in Google Maps) to classify each of 36 candidate shelters based on three characteristics within a 50 m buffer: (1) number of trees (≤ 10 or >10), (2) presence of shrubs (present or not), and (3) amount of vegetative cover (< 1/3 or ≥ 1/3 of the buffer zone occupied by vegetation). We assessed these characteristics in-person at two shelters for which Street View was not available, and we also ground-truthed shelter classifications after the onset of surveys and noted any discrepancies from the initial classifications. We used a stratified random sampling scheme to select a subsample of 16 shelters, two each from each possible class combination. Two of the shelters were rendered structurally incomplete or inaccessible early in the study due to construction; therefore, we replaced them with two randomly selected shelters from the same category as the original bus shelters, where possible. We also opportunistically added three shelters to increase sample size, and had to later remove one shelter due to construction activity, leaving us with a sample size of 18 shelters (Table 1). Six of the monitored shelters were on the Oklahoma State University (OSU) campus, while the remaining 12 shelters were along roadways in residential and commercial areas ranging from Stillwater’s downtown area to the outlying edges of the city.

**Table 1:** Variables measured for assessment of bird collision correlates at bus shelters in Stillwater, Oklahoma, USA.

Variable	Description
GlassArea	Total area (m^2^) of clear glass in shelter exterior
UrbanArea	Area (m^2^) of urban cover within 50-m buffer zone
ForestArea	Area (m^2^) of tree and shrub cover within 50-m buffer zone
LawnArea	Area (m^2^) of grass cover within 50-m buffer zone
ShrubDistance	Distance (m) from the bus shelter to the closest shrub
TreeDistance	Distance (m) from the bus shelter to the closest tree
TreeNumber	Number of trees present within 50-m buffer zone

### Carcass surveys

We surveyed for bird collisions twice a week from 4 May to 30 September 2016, between 10:00 a.m. and 2:00 p.m. because bird collisions with low structures often occur during early and late morning hours [18,10]. We adapted our survey procedure from Hager and Cosentino’s standardized protocol for collision monitoring at buildings [19]. Surveyors walked slowly around the entire perimeter of each shelter, searching the ground for intact carcasses or bird remains within two meters of all interior and exterior surfaces. All bus shelter surfaces were also inspected for feather spots, smudges, and silhouettes (hereafter, collectively “collision evidence”). Observations of collision evidence found on a shelter façade directly above a carcass were assumed to correspond with it and were not counted as separate collision incidents. Surveyors recorded the date, time, shelter number, and number and description of carcasses and collision evidence during each survey. To reduce bias associated with directional perception (e.g., objects being obscured when viewed from certain angles), we altered the survey direction (clockwise and counterclockwise) between survey days. To avoid counting fatalities and collision evidence from before the study period or double counting new fatalities and evidence during the study period, we removed all carcasses and collision evidence 24 hours before the initial survey and upon discovery in subsequent surveys. All surveys were conducted by the authors.

Upon detecting a carcass, we took photographs of the carcass in the context of its surroundings, as well as close-up images of the dorsal, ventral, and lateral surfaces of the bird. Additionally, we recorded the species and a description of the specific carcass location relative to the bus shelter. To minimize disease transmission, we used plastic bags to avoid direct skin contact with carcasses, and we double-bagged all carcasses with an identification label in Ziploc bags for storage in a freezer. Several carcasses were left in place as part of a scavenger removal trial (see below). We photographed any collision evidence and noted its specific location on the bus shelter before removing it. We were unable to identify species for feathers found adhered to bus shelters because most of these cases consisted of only one or a few body feathers. Carcasses were collected under Scientific Collector’s Permits obtained through the U.S. Fish and Wildlife Service and the Oklahoma Department of Wildlife Conservation, and our survey protocol was approved by the Institutional Animal Care and Use Committee at Oklahoma State University.

### Potential correlates of collision rates

We characterized several potential predictors of bird collision rates, including the amount of glass on shelters, the proximity of shelters to different vegetation types, and the number of trees and proportion of different land cover types within 50 m. Land cover variables were derived in ArcGIS 10.2 [20] using an aerial imagery layer with 1-m resolution [21] and Environment for Visualizing Images (ENVI) software (version 5.3)[22], which we used to conduct a Maximum Likelihood Classification of land cover data into 4 classes (Forest, Lawn, Urban, and Water). We created 50 m buffers around each shelter, within which we calculated the area of each land cover type (variable names used hereafter: ForestArea, LawnArea, UrbanArea, WaterArea). Shrubs and trees were both classified as ForestArea, and LawnArea included both mowed and unmowed grass. We used the original aerial imagery to ground-truth our classifications in each buffer.

**Fig 1:**
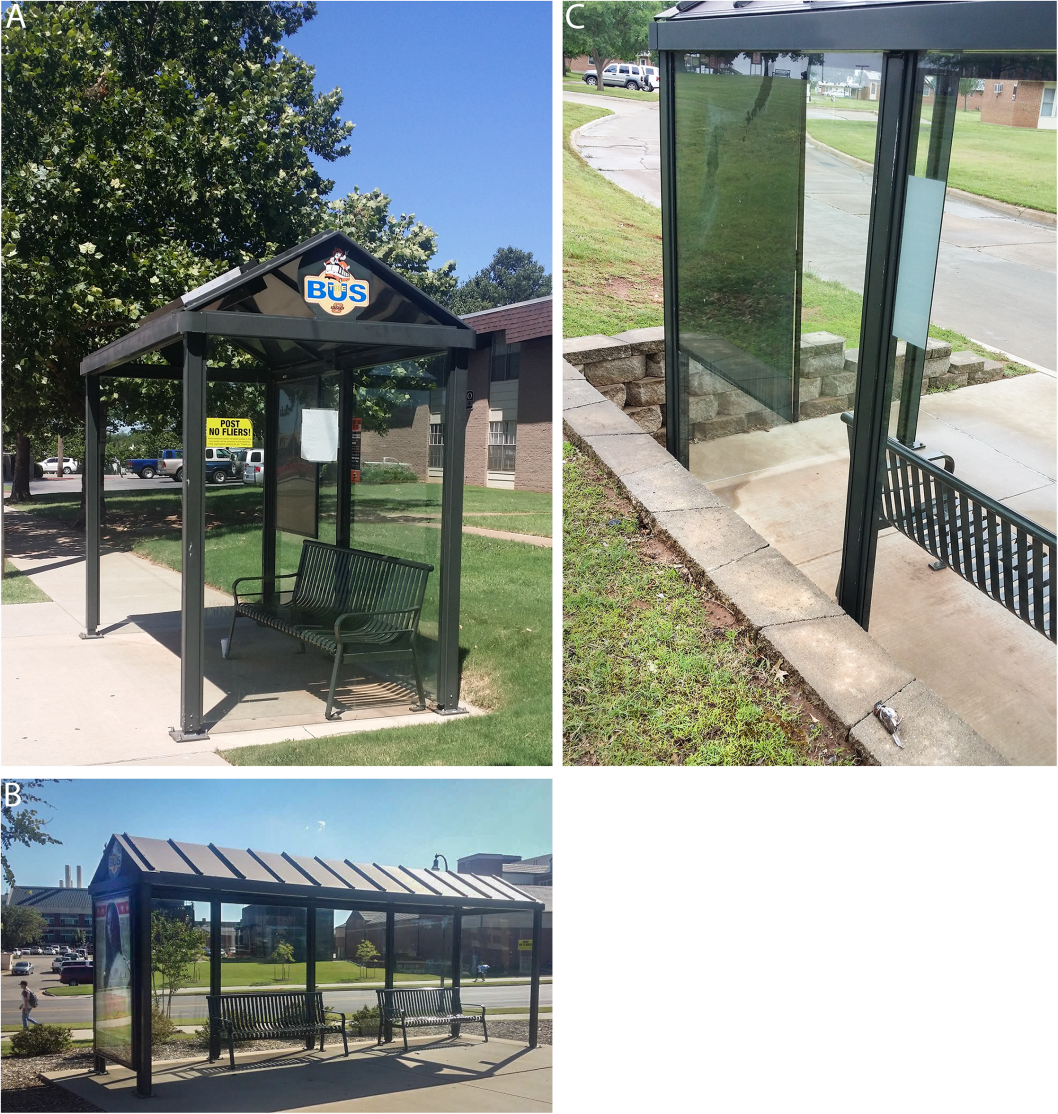
**Examples of typical bus shelters monitored in our study with four (A) and five (B) transparent glass panes, and a House Sparrow killed by collision with a four-paned bus shelter (C)**.

To characterize vegetation proximity variables, we used a tape measure to measure the distance of each shelter to the nearest tree (TreeDistance) and nearest shrub (ShrubDistance), taking the reading at the nearest branch or leaf. In cases where the most direct path between the shelter and vegetation was impeded by obstacles (e.g., buildings or cars), we used the Pythagorean Theorem to calculate distance. The number of trees within 50 m (TreeNumber) was determined using in-person visual counts at each shelter. Most bus shelters had a similar structure, consisting of 3–5 identical clear glass panes (Fig 1A, 1B and 1C). To estimate total glass area for each shelter (GlassArea), we multiplied the surface area of a standard pane by the number of panes, subtracting the area of any opaque coverings such as posters or appliqués. For one shelter that had panes of a non-standard design, all panes were measured separately.

### Experimental scavenger removal trials

Removal of carcasses by humans or animal scavengers can bias the findings of studies that are based on carcass detection [23,24]. To account for human removal of carcasses, we contacted OSU Transit Services and the City of Stillwater at the onset of the study to request that carcasses at bus shelters not be removed by maintenance personnel for the duration of the research. To estimate rate of carcass removal by scavengers, we monitored 11 carcasses daily until they were no longer detectable, with observations right censored at 30 days for the purposes of analyses. Eight carcasses, four each in June and September, were intentionally placed at randomly selected shelters. All placed carcasses were European Starlings (*Sturnus vulgaris*) that had been previously found as window collision fatalities, then collected and frozen, as part of a related research project. For the current study, all previously frozen carcasses were thawed 24 hours before placement, handled with a Ziploc bag to prevent contamination with human scent, and set within two meters of a randomly selected glass façade to simulate an actual window strike. Carcasses were placed on a variety of substrates, ranging from bare concrete to completely mulch- or grass-covered. The other three carcasses were found as collision victims at bus shelters during the current study and left in place after detection. These included a Brown Thrasher (*Toxostoma rufum*) (found on 5 Jun) and House Finch (*Haemorhous mexicanus*) (found on 29 Sep) detected during formal collision surveys and an American Robin (*Turdus migratorius*) (found on 30 May) discovered incidentally at a bus shelter not included in formal monitoring surveys.

### Analysis

All statistical analyses were conducted in program R [25] with the RStudio interface [26]. We used linear regression analyses to assess relationships between potential explanatory variables (Table 1) and two separate response variables, including: (1) numbers of carcasses found, and (2) numbers of observations of collision evidence. We ranked alternative models using Akaike’s Information Criterion corrected for small samples (AICc). We conducted model selection for all single-variable models, one each for each predictor variable, and then used the resulting ranking to create additive bivariate models containing combinations of the strongest single variables. Our set of candidate models for each response variable included additive two-variable models, high-ranking single variable models, and null models. Explanatory variables that expressed collinearity (|r^2^| > 0.70) were not included in the same model. Based on this criterion, UrbanArea was not included in models with LawnArea or ForestArea due to its collinearity with both of these variables. Additionally, the number of standard-sized glass panes was not included due to its collinearity with GlassArea. For both the carcass and collision evidence analyses, we also conducted model selection separately for two data sets: (1) all 18 monitored bus shelters, and (2) a subset of 15 shelters that excluded two randomly selected shelters from a cluster of three with overlapping 50 m buffers and one shelter that was a potential outlier due to an exceptionally high carcass count—as well as being the only shelter with a vegetative class (LawnArea) exceededing UrbanArea as the primary land cover. When ranking alternative candidate models, we considered models to receive strong support when they outperformed the null model and had a ΔAICc value <2. We considered variables to be uninformative if they only appeared in additive models that were ≤2 units from the best model but only differed from the best model by one parameter [27]. Because ΔAICc values only reflect relative model performance and are not indicative of strength of relationship, we further examined point estimates and standard errors of coefficients for variables from strongly supported models.

We accounted for scavenger removal of carcasses using the R package “carcass” [28] to estimate carcass persistence, and we used this value to generate adjusted estimates of mortality for our study area. We used the “persistence.prob” function to estimate carcass persistence and assumed constant persistence probability over time due to a small sample size [28]. Persistence estimates were then used in the “phuso” function [29] with perfect observer detection and a search interval of 3.5 days [28]. We assumed observer detection to be 100% because the search areas were small (approximately 36–70 m^2^) and had high visibility with contrasting substrates (often concrete) and few obscuring structures (e.g., shrubs, ground cover, or garbage bins). As searches were biweekly, the actual interval was either 3 or 4 days, which we averaged to 3.5 days. We divided the total number of observed carcasses by the resultant carcass persistence probability to estimate the minimum number of birds killed from May to September by colliding with the 18 monitored bus shelters. Finally, we conducted a simple extrapolation to estimate the total number of birds killed across all bus shelters in the city of Stillwater from May to September (estimated number killed per shelter per month X 36 shelters X 5 months) and for the entire calendar year (estimated number killed per shelter per month X 36 shelters X 12 months).

## Results

**Fig 2:**
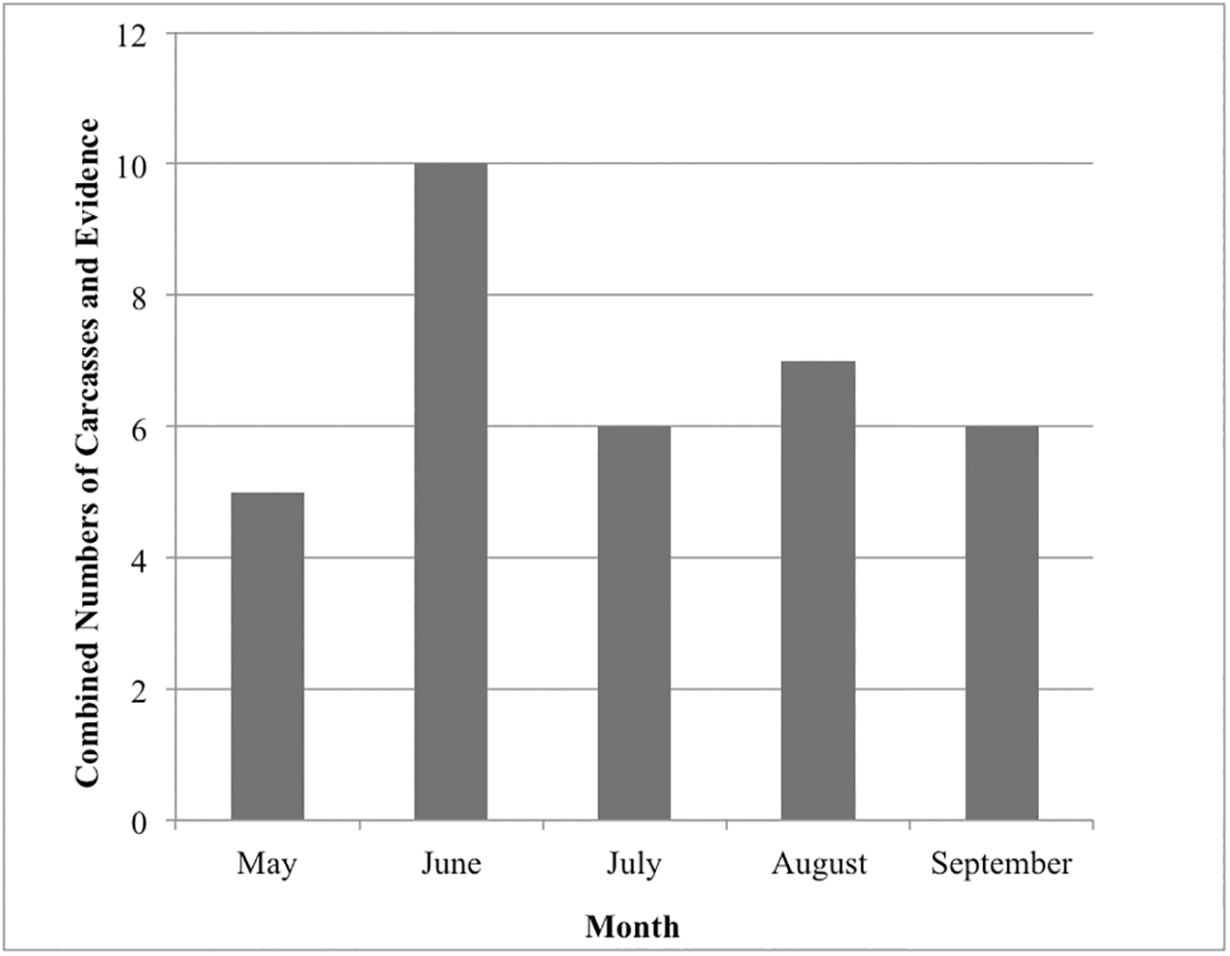
Total number of inferred collisions (carcasses plus incidents of collision evidence) per month at 18 monitored bus shelters.

Carcasses or other collision evidence were observed at 11 (61%) of the 18 bus shelters surveyed with an average of 0.7 carcasses per shelter (range = 0–4). We documented a total of 13 carcasses representing seven bird species (Table 2) and 21 observations of collision evidence on glass façades. House Finches and House Sparrows (*Passer domesticus*) were the most frequently found collision victims, accounting for 46% of carcasses. The month with the highest number of total inferred collisions, including both carcasses and collision evidence, was June (n = 10) (Fig 2). The greatest number of collision fatalities occurred in June (n = 4), July (n = 4), and September (n = 4), and collision evidence was found in every month surveyed. All carcasses except one were found on the exterior of bus shelters, but 62% of collision evidence observations were on an interior façade of bus shelters.

**Table 2:** Number and species of bird carcasses found at 18 monitored bus shelters in Stillwater, Oklahoma.

Species	Scientific Name	Carcasses^a^
House Sparrow	*Passer domesticus*	3
House Finch	*Haemorhous mexicanus*	3
Northern Mockingbird	*Mimus polyglottos*	2
Northern Cardinal	*Cardinalis cardinalis*	2
Brown Thrasher	*Toxostoma rufum*	1
Cooper’s Hawk	*Accipiter cooperii*	1
Scissor-Tailed Flycatcher	*Tyrannus forficatus*	1

^**a**^ Total number of intact carcasses of each species found across all surveys.

The modeling results for the assessment of potential correlates of mortality showed some similarities between the full data set of all 18 shelters and the reduced data set of 15 shelters. For the analysis of bird carcasses, the two best-supported models for both data sets were the single variable model containing LawnArea and the additive model containing LawnArea and GlassArea (Tables 3 and 4). For the reduced data set, the two-variable model with LawnArea and TreeNumber was also a high-ranking model, but TreeNumber was uninformative based on our above definition. Further assessment of model coefficients for variables from strongly supported models illustrated that collision rate was positively related to both GlassArea (β ± Standard Error [SE] = 0.207±0.104 for the full data set; 0.121±0.079 for the reduced data set) and LawnArea (β ± SE = 0.0007±0.0002 for the full data set; 0.0003±0.0002 for the reduced data set).

**Table 3:** Top five models based on Akaike’s Information Criteria (corrected for small sample size) in assessment of correlates of collision mortality, as inferred from direct observations of carcasses at 18 monitored bus shelters in Stillwater, Oklahoma.

Model	AICc^a^	ΔAICc^b^	K^c^	Weight^d^
GlassArea+LawnArea	52.0	0.0	4	0.4857
Lawn Area	52.9	0.9	3	0.3172
GlassArea+UrbanArea	54.9	2.9	4	0.1155
UrbanArea	57.7	5.7	3	0.0286
GlassArea	57.7	5.7	3	0.0276

^a^ AICc- AIC value, corrected for small sample size

^b^**Δ**AICc- relative difference in AICc value from the best-supported model

^c^ K- number of parameters

^d^ Weight- weight of corresponding AICc value

**Table 4:** Top five models based on Akaike’s Information Criteria (corrected for small sample size) in assessment of correlates of collision mortality, as inferred from direct observations of carcasses from a reduced dataset of 15 monitored bus shelters in Stillwater, Oklahoma.

Model	AICc^a^	ΔAICc^b^	K^c^	Weight^d^
LawnArea	29.6	0.0	3	0.282
GlassArea+LawnArea	30.4	0.8	4	0.187
GlassArea	30.7	1.1	3	0.162
LawnArea+TreeNumber^e^	30.9	1.3	4	0.144
Null	31.9	2.3	2	0.089

^a^ AICc- AIC value, corrected for small sample size

^b^ **Δ**AICc- relative difference in AICc value from the best-supported model

^c^ K- number of parameters

^d^ Weight- weight of corresponding AICc value

^e^ Based on our above definition, TreeNumber is an uninformative variable.

For the analysis of collision evidence, the single variable model containing GlassArea received the greatest support for both data sets (Tables 5 and 6); however, this model was only 0.9 above the null model for the analysis of the full data set, indicating relatively weak support. For the reduced data set, the two-variable model including GlassArea and TreeDistance also received strong support, but TreeDistance was an uninformative variable. Based on the top model from the reduced data set, the number of observations of collision evidence was positively related to GlassArea (β ± SE = 0.662±0.221).

**Table 5:** Top five models based on Akaike’s Information Criteria (corrected for small sample size) in assessment of correlates of collision mortality, as inferred from direct observations of collision evidence at 18 monitored bus shelters in Stillwater, Oklahoma.

Model	AICc^a^	ΔAICc^b^	K^c^	Weight^d^
GlassArea	74.2	0.0	3	0.346
Null	75.1	0.9	2	0.219
ForestArea	76.4	2.2	3	0.115
GlassArea+ForestArea	77.1	2.9	4	0.082
GlassArea+LawnArea	77.2	2.9	4	0.080

^a^ AICc- AIC value, corrected for small sample size

^b^ **Δ**AICc- relative difference in AICc value from the best-supported model

^c^ K- number of parameters

^d^ Weight- weight of corresponding AICc value

**Table 6:** Top five models based on Akaike’s Information Criterion (corrected for small sample size) in assessment of correlates of collision mortality, as inferred from direct observations of collision evidence at a reduced dataset of 15 monitored bus shelters in Stillwater, Oklahoma.

Model	AICc^a^	ΔAICc^b^	K^c^	Weight^d^
GlassArea	60.2	0.0	3	0.415
GlassArea+TreeDistance^e^	61.4	1.1	4	0.237
GlassArea+LawnArea	62.3	2.1	4	0.147
GlassArea+ForestArea	62.8	2.6	4	0.115
Null	65.2	5.0	2	0.034

^a^ AICc- AIC value, corrected for small sample size

^b^**Δ**AICc- relative difference in AICc value from the best-supported model

^c^ K- number of parameters

^d^ Weight- weight of corresponding AICc value

^e^ Based on our above definition, TreeDistance is an uninformative variable.

The average length of time carcasses remained until they were no longer detectable in our experimental scavenger removal trials was 7.25 days. The estimated carcass persistence probability between surveys was 0.758; when applied to our raw carcass count this probability produced a minimum estimate of 17.16 total carcasses occurring at monitored bus shelters during the study period. Assuming constant mortality across locations, we estimate a minimum 34.3 birds are killed between May and September at the 36 bus shelters in the city of Stillwater. This would equate to a minimum of 82.4 birds killed per year, assuming constant mortality throughout the year, an assumption that requires further validation with field research from October to April.

## Discussion

This study, the first to describe the phenomenon of bird collisions with glass bus shelters, illustrates that collisions occur throughout the late spring to early fall period and affect a variety of bird species, including both urban year-round residents (e.g., House Sparrows, House Finches, and Northern Cardinals [*Cardinalis cardinalis*]) and summer resident migratory species (Scissor-tailed Flycatcher [*Tyrannus forficatus*]). We found that fatal collisions and collision evidence were both positively related to the amount of glass on bus shelters, and additionally, that fatal collisions were positively related to the area of lawn within 50 m. Based on experimental trials, we documented a relatively low rate of scavenger removal of carcasses, information we used to generate a minimum estimate of roughly 34 birds killed by bus shelter collisions from May to September in our study area.

Our finding that bus shelters with a greater area of glass caused more collisions is consistent with studies showing that buildings with large expanses of glass are more hazardous to birds [30,8,31]. This result also provides further support for Hager et al.’s “window area hypothesis” that states that the amount of sheet glass on building exteriors is proportional to the number of fatal bird collisions that occur [12]. The effect of large amounts of glass may pose an even greater challenge to bird perception at bus shelters because the glass façades of shelters typically appear directly adjacent to sky or surrounding vegetation and lack the cues that likely help birds avoid glass collisions at buildings (e.g., the building profile and the opaque surfaces immediately surrounding windows). Additionally, the three-sided design of bus shelters may increase the frequency with which bird collisions occur due to initial entrapment within the structure. We directly observed one such entrapment incident where a juvenile robin was found perched on a bench inside the shelter and repeatedly collided with the transparent rear façade as it attempted to exit (however, the bird did not reach a high enough speed to cause severe injury). Our results provide evidence that collisions due to entrapment occur frequently at bus shelters; over half of our observations of collision evidence were on interior façades.

The relationship between glass area and the number of observations of collision evidence is perhaps not surprising because the various forms of collision evidence occur directly on façades and were only found on glass surfaces. While collision evidence provides clear indication that a bird strike has occurred, the outcomes of these collisions are unknown. Collision evidence could represent a non-fatal collision, a delayed fatality event whereby a bird dies after exiting the survey area, or a fatally killed or severely injured bird removed from the survey area by a scavenger prior to the subsequent survey. Thus, while monitoring for indirect collision evidence can contribute to understanding the frequency of total collisions at bus shelters, such evidence has limited use for estimating mortality. Additionally, Klem [30] found that one fourth of bird strikes at windows left no lasting evidence after a 24-hour period, and in our study, a single incident involving three collision fatalities at one shelter was only accompanied by a single feather mark. Therefore, counts of collision evidence likely substantially underestimate collision events.

Vegetation features surrounding buildings have been shown to influence bird-window collision rates, as birds collide when they fly towards perceived vegetation in reflected or transparent glass [10,11,8,30]. However, none of the vegetation features we assessed, including the number of trees within 50 m and the proximity of surrounding trees and shrubs, received support in predicting collision rates. One possible explanation for this negative result is that our sample of bus shelters did not capture sufficient variation in vegetation features to demonstrate an effect of nearby trees and shrubs. Further research that samples along a broader gradient of urbanization, including with a broader diversity of vegetation amounts and proximities, could provide further insight into the effect of vegetation on bird-shelter collisions.

Of the land cover variables we assessed, only the area of lawn within 50 m of bus shelters received support in predicting collision mortality. Lawn is the dominant vegetation cover type in our study area, a factor that may partially explain our findings because grassy areas provide foraging opportunities for many birds in urban areas, including the species found most frequently in our study (e.g., House Sparrows and House Finches) which is consistent with observations by Cusa et al. [31]. Another possible explanation for the correlation between fatal collisions and lawn area is that birds flying in open lawn areas might attain higher speeds than in areas with taller vegetation or human structures, and this may result in a higher probability of collisions being fatal. Further support for this explanation is provided by our finding that LawnArea was not a predictor of collision evidence, which ostensibly captured many non-fatal collisions. Although lawn area was the most prevalent vegetation cover type in our study area, impervious surface (e.g., roads, parking lots, and buildings) was the dominant land cover type overall. Buildings in areas with greater urbanization are associated with fewer collisions [12], likely because they lack green space and the associated resources to attract large numbers of birds [15]; however, we found no correlation in our study between bird collisions and area of urban land cover.

Whereas the bird species most vulnerable to building collisions are primarily long-distance Neotropical migrants [32,11,8,27,6], the majority of identifiable collision victims in our study were year-round resident urban-adapted species, including a non-native invasive species (House Sparrow), a human-introduced species native to western North America (House Finch), and native resident species (Northern Mockingbird and Northern Cardinal). Although we found three migratory species (Cooper’s Hawk, Brown Thrasher, and Scissor-tailed Flycatcher), we did not document any of the migratory species previously identified as highly vulnerable to building collisions [6], despite documenting many of these and other migratory species in concurrent research on bird-building collisions in the same study area (CSR and SRL unpublished data). Incidental observations of fatal bus shelter collisions in the same study area before and after the current standardized research (CMB, CSR, and SRL unpublished data) also illustrated a mix of urban-adapted and migratory species, but with 71% (12 of 17) of observations representing native species, including Western Kingbird (*Tyrannus verticalus*), American Robin, Cedar Waxwing (*Bombycilla cedrorum*), Northern Mockingbird, Northern Cardinal, and Dark-eyed Junco (*Junco hyemalis*). Because our sample size of bird carcasses is somewhat limited, further research is required to confirm the degree to which species of conservation concern are impacted and whether the species composition of collision victims indeed varies between buildings and bus shelters. However, we speculate that at least two major differences between buildings and bus shelters could result in Neotropical migrants being regularly killed at buildings but not at bus shelters, including: (1) differences in vegetation, with ornamental vegetation and landscaping that attracts migratory species generally more abundant near buildings than bus shelters and (2) differences in attraction mechanisms, with buildings, but not bus shelters, having external lighting that can attract and confuse nocturnally migrating Neotropical migrant species.

Several limitations may have influenced the results of our study. Our small sample size made it difficult to assess the interacting effects of multiple variables; monitoring a larger sample of bus shelters would provide further insight regarding any interactive effects of vegetation, land cover, and bus shelter characteristics as drivers of collisions. The search interval we employed (3–4 days between surveys) resulted in some carcasses being removed between surveys. We accounted for this bias in our estimate of mortality for the entire study area but not for the assessment of collision correlates because there were not enough trial replicates to generate separate scavenger removal estimates for each bus shelter. Nonetheless, our experimental trials revealed that the carcass removal rate was low, with an average carcass persistence time of 7.25 days, and we have no evidence to suggest that removal rates differed among shelters with different characteristics. Additionally, we did not account for searcher detection bias (i.e., when surveyors fail to detect all carcasses present on surveys, [33]); however, our assumption of 100% detection is more likely to be met at bus shelters than at larger structures due to the small search area and the surrounding surfaces (i.e., primarily pavement) on which carcasses are easily visible.

In addition to the above-described research needs, future studies of bird collisions at bus shelters should consider taking a broader comparative approach across multiple municipalities. Such an approach would both lead to a greater understanding of the anthropogenic forces which shape urban biota across cities [34, 35] and allow for a more nuanced assessment of the effect of varying land cover types and landscape patterns on collision rates. This approach would also result in a broader variety of bus shelter designs being captured, including those of varying sizes, heights, and orientations relative to surrounding vegetation and roadways, and with different types of glass. Along with additional research at a large number of study sites, obtaining an estimate of the overall number of bus shelters across North America is necessary for generating regional or national estimates of bird collision mortality from bus shelter collisions. With a rough minimum estimate of 34 fatalities across a five-month period in a single small city, the total number of bus shelter-related bird deaths across the continent could be substantial. However, as described above, the relative representation of different species, and thus the likelihood of population impacts for species of conservation concern, requires further research.

Our results suggest that constructing bus shelters with a smaller amount of glass surface area is likely to be the most effective way to reduce avian collision mortality, paralleling Kahle et al.’s similar finding for building windows [36]. However, in cases where glass panes are required due to safety considerations or where glass shelters are already in place, retrofitting to reduce mortality may still be possible. Notably, several U.S. cities are already retrofitting glass bus shelters—either to directly address bird collisions or to reduce vandalism—in ways that should make shelters more visible to birds. For example, shelters in San Francisco, California, and Minneapolis, Minnesota employ frit patterns on glass façades [37,38]. Additionally, following several incidental reports of fatal bird strikes, bus shelters at the National Renewable Energy Laboratory in Golden, Colorado were retrofitted with an external window film and have since had no reported collisions (Thomas Ryon, pers. comm.). Some European cities have also introduced creative shelter designs resulting in bird-friendly shelters, with examples in Munich, Germany, and in Basel and Zurich, Switzerland [39]. Explicitly considering bird collision risk in the design of new bus shelters and retrofits of existing bus shelters will ensure the greatest reductions in mortality, and several building design guides that have been developed to address bird-window collision risk should also apply for bus shelters [40,41].

Given this growing interest in reducing collisions, bus shelters have the potential to serve as models for research that field tests the efficacy of bird collision deterrents. Many products are commercially marketed to reduce bird-glass collisions, including glass with inlaid patterns, as well as externally applied tapes, films, gels, and decals; however, research into the effectiveness of such products has to date been confined to lab tests in experimental flight tunnels [42] or to experimentally-placed panes of glass suspended in open habitat [30]. Both of these approaches have provided valuable insights into reducing bird mortality from glass collisions; however, in-field testing of bird deterrent products on building windows is also necessary to realistically understand avian responses to and effectiveness of collision deterrent techniques. Because of the logistic constraints of applying test products to a large number of windows at a large number of buildings, bus shelters may provide an appealing alternative for beta-testing of potential collision deterrent technologies.

Notably, the implications of our research extend to any location where glass bus shelters are found. With increasing urbanization, cities continue to expand outward, with new bus shelters erected and existing shelters replaced with new glass structures. While many threats affecting birds are indirect (e.g., habitat loss and climate change) and thus difficult to address, bird fatalities resulting from collisions, including collisions with bus shelters, have comparatively direct effects with identifiable solutions. Shelters are one aspect of the multi-faceted bird-glass collision issue that directly results in a tremendous number of avian fatalities each year. Nonetheless, understanding the collective effect of all manmade structures that pose a hazard to birds is crucial both to assessing and reversing cumulative impacts of mortality sources on avian populations and to generating a comprehensive understanding of anthropogenic impacts in urban landscapes.
